# A Strategy for Developing an HIV Vaccine

**DOI:** 10.1371/journal.pmed.0020035

**Published:** 2005-01-18

**Authors:** The PLoS Medicine Editors

## Abstract

The newly published strategic plan for developing an HIV vaccine is crucially important, say the PLoS Medicine editors, but it must be followed by clear milestones and a process for monitoring progress

## Teaser

The new global plan is exciting, but now needs clear milestones

In 1997, United States President Bill Clinton announced the challenge to develop an AIDS vaccine by 2007. Since 1997, the AIDS Vaccine Advocacy Coalition (AVAC) has published annual reports on the global status of the effort to meet Clinton's deadline. Last year's report, entitled “AIDS Vaccine Trials—Getting the Global House in Order,” officially ends the countdown. Saying that “we are on a long term mission,” AVAC concludes that there will not be a safe and efficient vaccine in 2007, and that we need to “focus on the long haul and set an agenda for sustained and sustainable action that stretches well beyond 2007.” It is not that there are no vaccine candidates in clinical trials, but there is little hope that any of the current candidates will turn out to be a cheap and safe vaccine that affords long-term protection.

Among notable developments over the past 12 months, the AVAC report highlights the Global HIV/AIDS Vaccine Enterprise as an effort to improve coordination within the AIDS vaccine field. The Enterprise was announced in June 2003 and now shares its scientific strategic plan with everyone affected by the AIDS pandemic—that is, all of us—by publishing it in *PLoS Medicine* (DOI: 10.1371/journal.pmed.0020025).

In its plan the Enterprise presents itself as a global endeavor and emphasizes the need for integration and capacity building around the world. It is not “a discrete organization with a pool of money” but a “coordinating group of individual funding agencies that will support specific areas of research using their own mechanisms, according to their own practices and policies, and following the Enterprise's principles.” These principles include collaboration, standardization, and coordination among international researchers and agencies. The plan focuses on specific scientific roadblocks that need to be overcome, but also looks ahead and mentions the need to build capacity for product manufacturing and clinical trials, and to address regulatory issues.

These are noble goals, and the fact that they are stipulated jointly by many of the leaders in the field will generate excitement and expectations, even though much of what is said has been said before. The plan stresses collaboration and coordination; there are clear benefits from a concerted effort. But might a level of competition, rather than collaboration, be healthy, and, if so, what level of competition would work best? The Enterprise members seem to have wrestled with that question. The plan mentions an “appropriate balance between productive competition and effective collaboration,” and suggests that certain incentives could be provided by “the funders with greatest flexibility.” As long as it remains unclear where scientific breakthroughs will come from, diversity and flexibility should be encouraged and not stifled. David Ho, in his Perspective on the plan (DOI: 10.1371/journal.pmed.0020036), mentions the danger of “group think,” and the Enterprise must not fall into that trap.

Notably absent from this initial plan is any mention of a timeline or milestones. The remit of the plan's authors was not to prescribe specific research but “to stimulate both researchers and funders to explore new, more collaborative, cooperative, and transparent approaches…in addition to continuing the productive, high-quality approaches already underway.” However, without a timeline, the plan fails to convey a sense of urgency. This is problematic, as any delays in developing a vaccine will increase the burden from HIV/AIDS in the parts of the world that can least afford it. To accelerate vaccine development, the plan urgently needs to be supplemented with a list of specific tasks, responsible individuals, necessary resources, and allocated time.

The next document from the Enterprise must provide specifics on project management, although one problem with putting a time frame on HIV vaccine development is a fundamental one: we do not know whether it is actually possible to develop a safe and effective vaccine. (One assumes the Enterprise members agree, though there is no explicit acknowledgement of this uncertainty in the plan.) Moreover, provided it can be done, it is impossible to predict when the necessary scientific advances will happen. That said, without a list of specific projects, project leaders, and a time frame for achieving or at least evaluating specific goals, it will be impossible to define success and failure, review progress, and assure internal and external accountability.

There is another reason why a best-guess timeline is essential: realistic expectations about an AIDS vaccine would stress the urgency of combating the AIDS pandemic over the next decade—and maybe longer—in the absence of an effective vaccine. The potential benefits of a vaccine cannot be overestimated, and its development has to be one top priority for the global scientific community. But its success cannot be taken for granted and will come too late for millions. Therefore, parallel efforts to prevent or reduce transmission and to treat infected individuals need to be accelerated now.

The Enterprise's plan should be hailed as a crucially important outline for vaccine development, but the goodwill surrounding it won't last unless it is quickly followed up with a set of milestones, and a transparent process by which progress will be measured and course corrections implemented.

